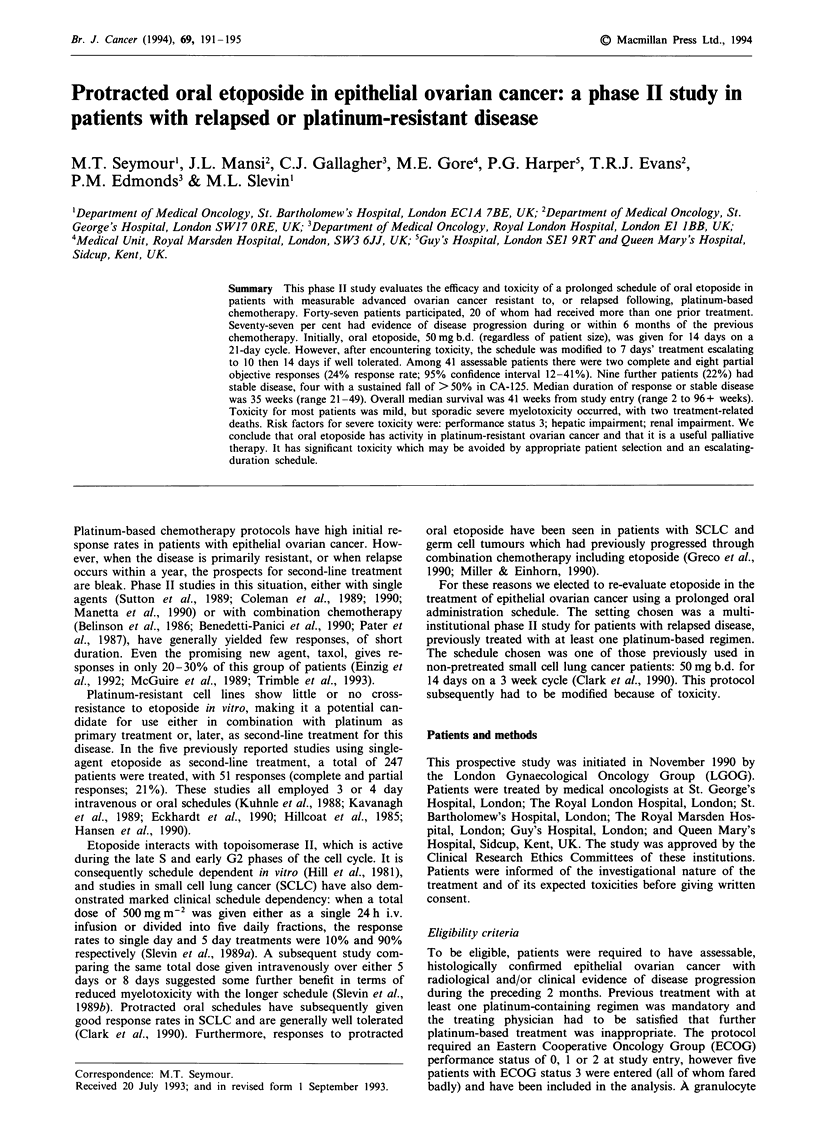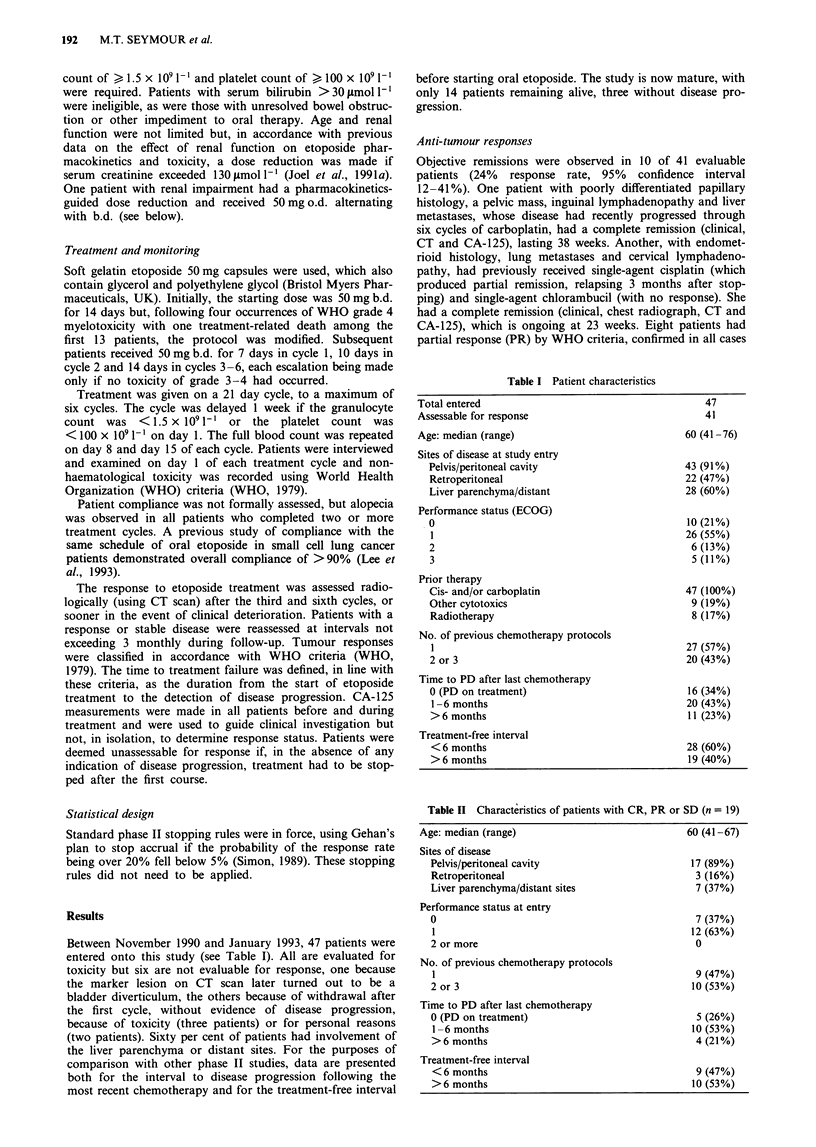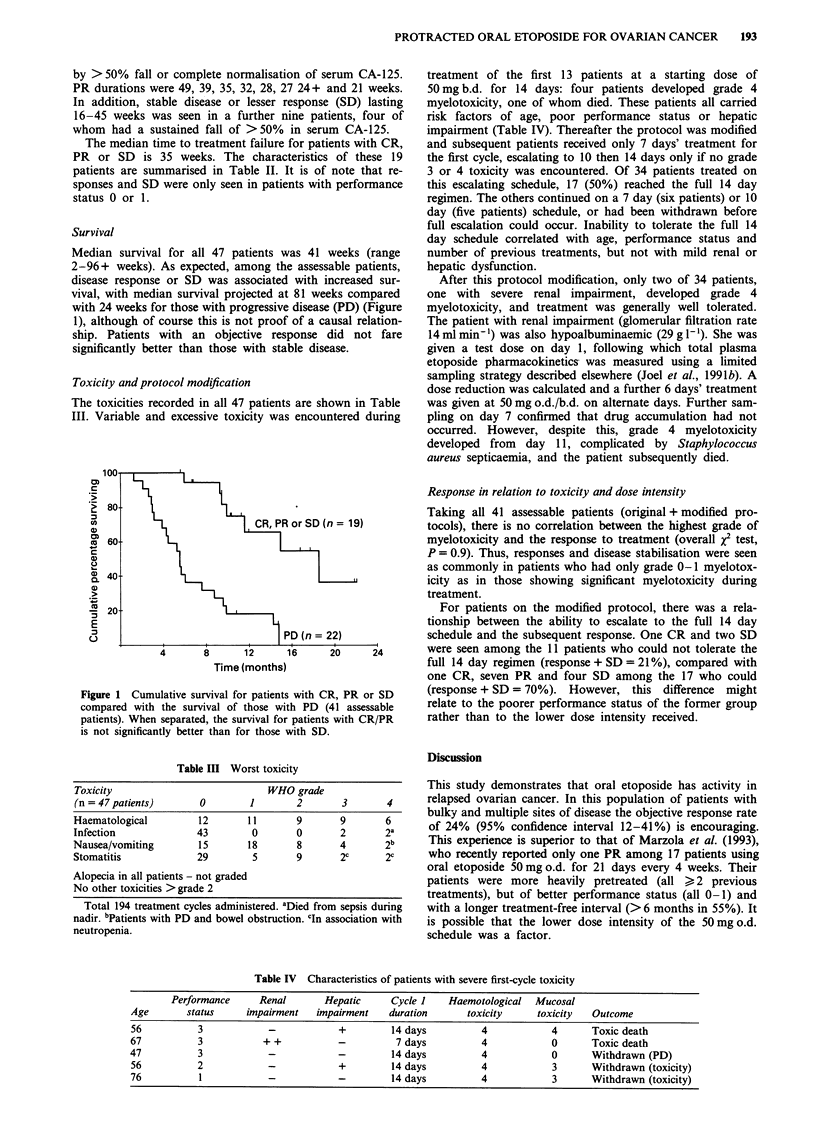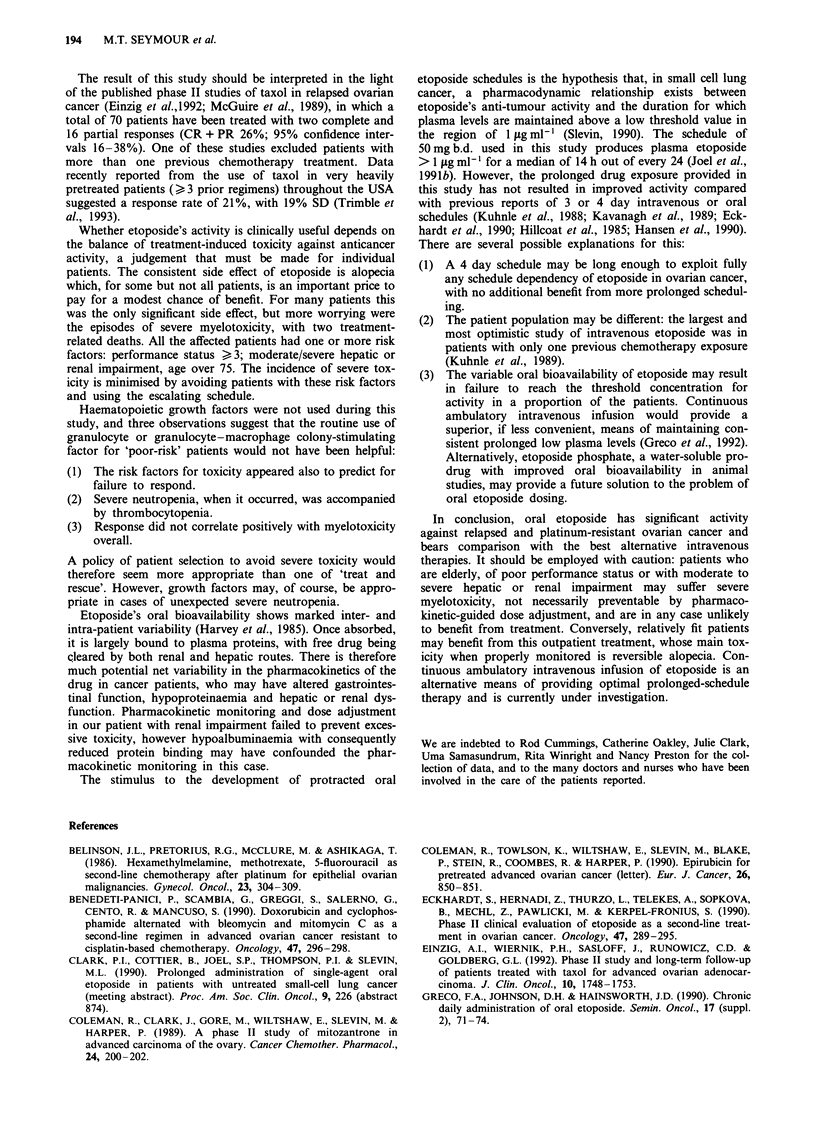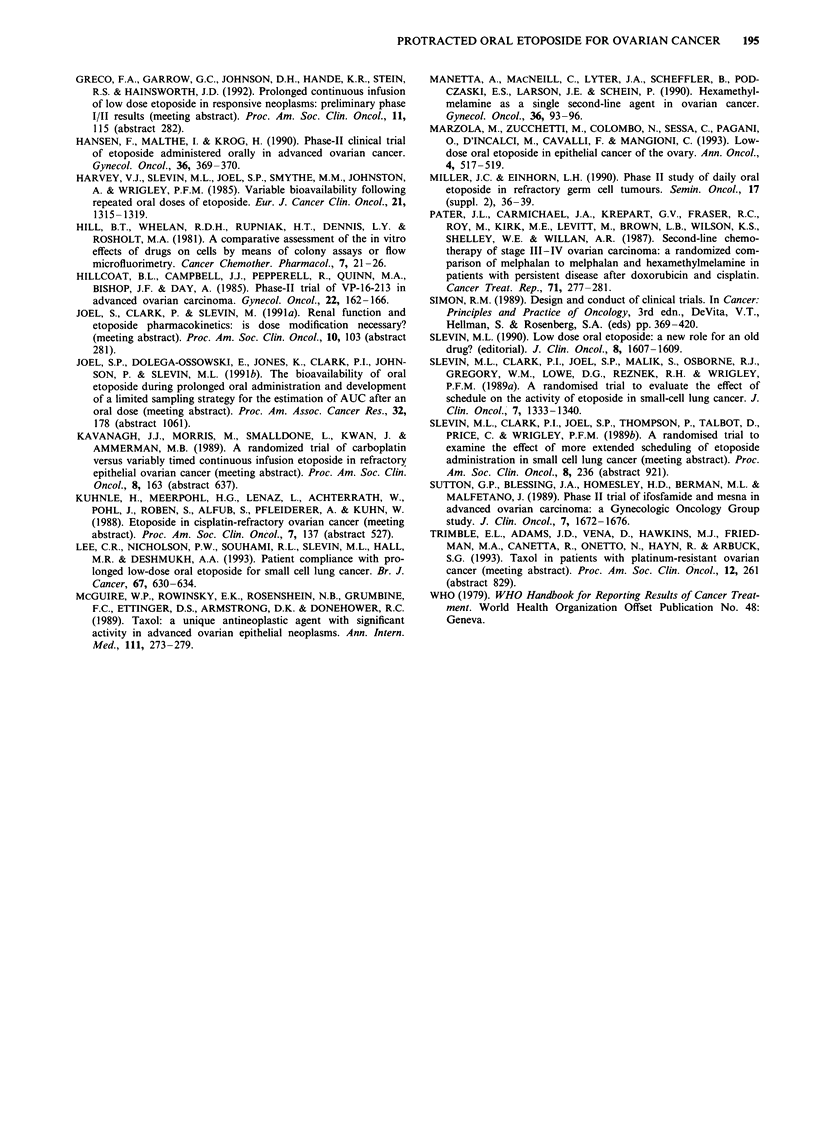# Protracted oral etoposide in epithelial ovarian cancer: a phase II study in patients with relapsed or platinum-resistant disease.

**DOI:** 10.1038/bjc.1994.33

**Published:** 1994-01

**Authors:** M. T. Seymour, J. L. Mansi, C. J. Gallagher, M. E. Gore, P. G. Harper, T. R. Evans, P. M. Edmonds, M. L. Slevin

**Affiliations:** Department of Medical Oncology, St. Bartholomew's Hospital, London, UK.

## Abstract

This phase II study evaluates the efficacy and toxicity of a prolonged schedule of oral etoposide in patients with measurable advanced ovarian cancer resistant to, or relapsed following, platinum-based chemotherapy. Forty-seven patients participated, 20 of whom had received more than one prior treatment. Seventy-seven per cent had evidence of disease progression during or within 6 months of the previous chemotherapy. Initially, oral etoposide, 50 mg b.d. (regardless of patient size), was given for 14 days on a 21-day cycle. However, after encountering toxicity, the schedule was modified to 7 days' treatment escalating to 10 then 14 days if well tolerated. Among 41 assessable patients there were two complete and eight partial objective responses (24% response rate; 95% confidence interval 12-41%). Nine further patients (22%) had stable disease, four with a sustained fall of > 50% in CA-125. Median duration of response or stable disease was 35 weeks (range 21-49). Overall median survival was 41 weeks from study entry (range 2 to 96+ weeks). Toxicity for most patients was mild, but sporadic severe myelotoxicity occurred, with two treatment-related deaths. Risk factors for severe toxicity were: performance status 3; hepatic impairment; renal impairment. We conclude that oral etoposide has activity in platinum-resistant ovarian cancer and that it is a useful palliative therapy. It has significant toxicity which may be avoided by appropriate patient selection and an escalating-duration schedule.


Br. J. Cancer (1994), 69, 191-195 © Macmillan Press Ltd., 1994

### Protracted oral etoposide in epithelial ovarian cancer: a phase II study in patients with relapsed or platinum-resistant disease

M.T. Seymour[1], J.L. Mansi[2], C.J. Gallagher[3], M.E. Gore[4], P.G. Harper[5], T.R.J. Evans[2], P.M. Edmonds[3] & M.L. Slevin[1]

[1]*Department of Medical Oncology, St. Bartholomew's Hospital, London EC1A 7BE, UK;* [2]*Department of Medical Oncology, St. George's Hospital, London SW17 0RE, UK;* [3]*Department of Medical Oncology, Royal London Hospital, London E1 1BB, UK;* [4]*Medical Unit, Royal Marsden Hospital, London, SW3 6JJ, UK;* [5]*Guy's Hospital, London SE1 9RT and Queen Mary's Hospital, Sidcup, Kent, UK.*

**Summary** This phase II study evaluates the efficacy and toxicity of a prolonged schedule of oral etoposide in patients with measurable advanced ovarian cancer resistant to, or relapsed following, platinum-based chemotherapy. Forty-seven patients participated, 20 of whom had received more than one prior treatment. Seventy-seven per cent had evidence of disease progression during or within 6 months of the previous chemotherapy. Initially, oral etoposide, 50 mg b.d. (regardless of patient size), was given for 14 days on a 21-day cycle. However, after encountering toxicity, the schedule was modified to 7 days' treatment escalating to 10 then 14 days if well tolerated. Among 41 assessable patients there were two complete and eight partial objective responses (24% response rate; 95% confidence interval 12–41%). Nine further patients (22%) had stable disease, four with a sustained fall of >50% in CA-125. Median duration of response or stable disease was 35 weeks (range 21–49). Overall median survival was 41 weeks from study entry (range 2 to 96+ weeks). Toxicity for most patients was mild, but sporadic severe myelotoxicity occurred, with two treatment-related deaths. Risk factors for severe toxicity were: performance status 3; hepatic impairment; renal impairment. We conclude that oral etoposide has activity in platinum-resistant ovarian cancer and that it is a useful palliative therapy. It has significant toxicity which may be avoided by appropriate patient selection and an escalating-duration schedule.

Platinum-based chemotherapy protocols have high initial response rates in patients with epithelial ovarian cancer. However, when the disease is primarily resistant, or when relapse occurs within a year, the prospects for second-line treatment are bleak. Phase II studies in this situation, either with single agents (Sutton *et al.*, 1989; Coleman *et al.*, 1989; 1990; Manetta *et al.*, 1990) or with combination chemotherapy (Belinson *et al.*, 1986; Benedetti-Panici *et al.*, 1990; Pater *et al.*, 1987), have generally yielded few responses, of short duration. Even the promising new agent, taxol, gives responses in only 20–30% of this group of patients (Einzig *et al.*, 1992; McGuire *et al.*, 1989; Trimble *et al.*, 1993).

Platinum-resistant cell lines show little or no cross-resistance to etoposide *in vitro*, making it a potential candidate for use either in combination with platinum as primary treatment or, later, as second-line treatment for this disease. In the five previously reported studies using single-agent etoposide as second-line treatment, a total of 247 patients were treated, with 51 responses (complete and partial responses; 21%). These studies all employed 3 or 4 day intravenous or oral schedules (Kuhnle *et al.*, 1988; Kavanagh *et al.*, 1989; Eckhardt *et al.*, 1990; Hillcoat *et al.*, 1985; Hansen *et al.*, 1990).

Etoposide interacts with topoisomerase II, which is active during the late S and early G2 phases of the cell cycle. It is consequently schedule dependent *in vitro* (Hill *et al.*, 1981), and studies in small cell lung cancer (SCLC) have also demonstrated marked clinical schedule dependency: when a total dose of 500 mg m$^{-2}$ was given either as a single 24 h i.v. infusion or divided into five daily fractions, the response rates to single day and 5 day treatments were 10% and 90% respectively (Slevin *et al.*, 1989*a*). A subsequent study comparing the same total dose given intravenously over either 5 days or 8 days suggested some further benefit in terms of reduced myelotoxicity with the longer schedule (Slevin *et al.*, 1989*b*). Protracted oral schedules have subsequently given good response rates in SCLC and are generally well tolerated (Clark *et al.*, 1990). Furthermore, responses to protracted oral etoposide have been seen in patients with SCLC and germ cell tumours which had previously progressed through combination chemotherapy including etoposide (Greco *et al.*, 1990; Miller & Einhorn, 1990).

For these reasons we elected to re-evaluate etoposide in the treatment of epithelial ovarian cancer using a prolonged oral administration schedule. The setting chosen was a multi-institutional phase II study for patients with relapsed disease, previously treated with at least one platinum-based regimen. The schedule chosen was one of those previously used in non-pretreated small cell lung cancer patients: 50 mg b.d. for 14 days on a 3 week cycle (Clark *et al.*, 1990). This protocol subsequently had to be modified because of toxicity.

### Patients and methods

This prospective study was initiated in November 1990 by the London Gynaecological Oncology Group (LGOG). Patients were treated by medical oncologists at St. George's Hospital, London; The Royal London Hospital, London; St. Bartholomew's Hospital, London; The Royal Marsden Hospital, London; Guy's Hospital, London; and Queen Mary's Hospital, Sidcup, Kent, UK. The study was approved by the Clinical Research Ethics Committees of these institutions. Patients were informed of the investigational nature of the treatment and of its expected toxicities before giving written consent.

### *Eligibility criteria*

To be eligible, patients were required to have assessable, histologically confirmed epithelial ovarian cancer with radiological and/or clinical evidence of disease progression during the preceding 2 months. Previous treatment with at least one platinum-containing regimen was mandatory and the treating physician had to be satisfied that further platinum-based treatment was inappropriate. The protocol required an Eastern Cooperative Oncology Group (ECOG) performance status of 0, 1 or 2 at study entry, however five patients with ECOG status 3 were entered (all of whom fared badly) and have been included in the analysis. A granulocyte

Correspondence: M.T. Seymour.
Received 20 July 1993; and in revised form 1 September 1993.

count of $\geqslant 1.5 \times 10^9 l^{-1}$ and platelet count of $\geqslant 100 \times 10^9 l^{-1}$ were required. Patients with serum bilirubin $> 30 \mu mol l^{-1}$ were ineligible, as were those with unresolved bowel obstruction or other impediment to oral therapy. Age and renal function were not limited but, in accordance with previous data on the effect of renal function on etoposide pharmacokinetics and toxicity, a dose reduction was made if serum creatinine exceeded $130 \mu mol l^{-1}$ (Joel et al., 1991a). One patient with renal impairment had a pharmacokinetics-guided dose reduction and received 50 mg o.d. alternating with b.d. (see below).

### *Treatment and monitoring*

Soft gelatin etoposide 50 mg capsules were used, which also contain glycerol and polyethylene glycol (Bristol Myers Pharmaceuticals, UK). Initially, the starting dose was 50 mg b.d. for 14 days but, following four occurrences of WHO grade 4 myelotoxicity with one treatment-related death among the first 13 patients, the protocol was modified. Subsequent patients received 50 mg b.d. for 7 days in cycle 1, 10 days in cycle 2 and 14 days in cycles 3–6, each escalation being made only if no toxicity of grade 3–4 had occurred.

Treatment was given on a 21 day cycle, to a maximum of six cycles. The cycle was delayed 1 week if the granulocyte count was $< 1.5 \times 10^9 l^{-1}$ or the platelet count was $< 100 \times 10^9 l^{-1}$ on day 1. The full blood count was repeated on day 8 and day 15 of each cycle. Patients were interviewed and examined on day 1 of each treatment cycle and non-haematological toxicity was recorded using World Health Organization (WHO) criteria (WHO, 1979).

Patient compliance was not formally assessed, but alopecia was observed in all patients who completed two or more treatment cycles. A previous study of compliance with the same schedule of oral etoposide in small cell lung cancer patients demonstrated overall compliance of $> 90\%$ (Lee et al., 1993).

The response to etoposide treatment was assessed radiologically (using CT scan) after the third and sixth cycles, or sooner in the event of clinical deterioration. Patients with a response or stable disease were reassessed at intervals not exceeding 3 monthly during follow-up. Tumour responses were classified in accordance with WHO criteria (WHO, 1979). The time to treatment failure was defined, in line with these criteria, as the duration from the start of etoposide treatment to the detection of disease progression. CA-125 measurements were made in all patients before and during treatment and were used to guide clinical investigation but not, in isolation, to determine response status. Patients were deemed unassessable for response if, in the absence of any indication of disease progression, treatment had to be stopped after the first course.

### *Statistical design*

Standard phase II stopping rules were in force, using Gehan's plan to stop accrual if the probability of the response rate being over 20% fell below 5% (Simon, 1989). These stopping rules did not need to be applied.

### Results

Between November 1990 and January 1993, 47 patients were entered onto this study (see Table I). All are evaluated for toxicity but six are not evaluable for response, one because the marker lesion on CT scan later turned out to be a bladder diverticulum, the others because of withdrawal after the first cycle, without evidence of disease progression, because of toxicity (three patients) or for personal reasons (two patients). Sixty per cent of patients had involvement of the liver parenchyma or distant sites. For the purposes of comparison with other phase II studies, data are presented both for the interval to disease progression following the most recent chemotherapy and for the treatment-free interval before starting oral etoposide. The study is now mature, with only 14 patients remaining alive, three without disease progression.

### *Anti-tumour responses*

Objective remissions were observed in 10 of 41 evaluable patients (24% response rate, 95% confidence interval 12–41%). One patient with poorly differentiated papillary histology, a pelvic mass, inguinal lymphadenopathy and liver metastases, whose disease had recently progressed through six cycles of carboplatin, had a complete remission (clinical, CT and CA-125), lasting 38 weeks. Another, with endometrioid histology, lung metastases and cervical lymphadenopathy, had previously received single-agent cisplatin (which produced partial remission, relapsing 3 months after stopping) and single-agent chlorambucil (with no response). She had a complete remission (clinical, chest radiograph, CT and CA-125), which is ongoing at 23 weeks. Eight patients had partial response (PR) by WHO criteria, confirmed in all cases

**Table I** Patient characteristics

| | |
|---|---|
| Total entered | 47 |
| Assessable for response | 41 |
| Age: median (range) | 60 (41–76) |
| Sites of disease at study entry | |
| Pelvis/peritoneal cavity | 43 (91%) |
| Retroperitoneal | 22 (47%) |
| Liver parenchyma/distant | 28 (60%) |
| Performance status (ECOG) | |
| 0 | 10 (21%) |
| 1 | 26 (55%) |
| 2 | 6 (13%) |
| 3 | 5 (11%) |
| Prior therapy | |
| Cis- and/or carboplatin | 47 (100%) |
| Other cytotoxics | 9 (19%) |
| Radiotherapy | 8 (17%) |
| No. of previous chemotherapy protocols | |
| 1 | 27 (57%) |
| 2 or 3 | 20 (43%) |
| Time to PD after last chemotherapy | |
| 0 (PD on treatment) | 16 (34%) |
| 1–6 months | 20 (43%) |
| >6 months | 11 (23%) |
| Treatment-free interval | |
| <6 months | 28 (60%) |
| >6 months | 19 (40%) |

**Table II** Characteristics of patients with CR, PR or SD ($n = 19$)

| | |
|---|---|
| Age: median (range) | 60 (41–67) |
| Sites of disease | |
| Pelvis/peritoneal cavity | 17 (89%) |
| Retroperitoneal | 3 (16%) |
| Liver parenchyma/distant sites | 7 (37%) |
| Performance status at entry | |
| 0 | 7 (37%) |
| 1 | 12 (63%) |
| 2 or more | 0 |
| No. of previous chemotherapy protocols | |
| 1 | 9 (47%) |
| 2 or 3 | 10 (53%) |
| Time to PD after last chemotherapy | |
| 0 (PD on treatment) | 5 (26%) |
| 1–6 months | 10 (53%) |
| >6 months | 4 (21%) |
| Treatment-free interval | |
| <6 months | 9 (47%) |
| >6 months | 10 (53%) |

by >50% fall or complete normalisation of serum CA-125. PR durations were 49, 39, 35, 32, 28, 27 24+ and 21 weeks. In addition, stable disease or lesser response (SD) lasting 16–45 weeks was seen in a further nine patients, four of whom had a sustained fall of >50% in serum CA-125.

The median time to treatment failure for patients with CR, PR or SD is 35 weeks. The characteristics of these 19 patients are summarised in Table II. It is of note that responses and SD were only seen in patients with performance status 0 or 1.

### *Survival*

Median survival for all 47 patients was 41 weeks (range 2–96+ weeks). As expected, among the assessable patients, disease response or SD was associated with increased survival, with median survival projected at 81 weeks compared with 24 weeks for those with progressive disease (PD) (Figure 1), although of course this is not proof of a causal relationship. Patients with an objective response did not fare significantly better than those with stable disease.

### *Toxicity and protocol modification*

The toxicities recorded in all 47 patients are shown in Table III. Variable and excessive toxicity was encountered during

**Figure 1** Cumulative survival for patients with CR, PR or SD compared with the survival of those with PD (41 assessable patients). When separated, the survival for patients with CR/PR is not significantly better than for those with SD.

**Table III** Worst toxicity

| *Toxicity* (n = *47 patients)* | WHO grade *0* | *1* | *2* | *3* | *4* |
|---|---|---|---|---|---|
| Haematological | 12 | 11 | 9 | 9 | 6 |
| Infection | 43 | 0 | 0 | 2 | 2[a] |
| Nausea/vomiting | 15 | 18 | 8 | 4 | 2[b] |
| Stomatitis | 29 | 5 | 9 | 2[c] | 2[c] |

Alopecia in all patients – not graded
No other toxicities >grade 2

Total 194 treatment cycles administered. [a]Died from sepsis during nadir. [b]Patients with PD and bowel obstruction. [c]In association with neutropenia.

treatment of the first 13 patients at a starting dose of 50 mg b.d. for 14 days: four patients developed grade 4 myelotoxicity, one of whom died. These patients all carried risk factors of age, poor performance status or hepatic impairment (Table IV). Thereafter the protocol was modified and subsequent patients received only 7 days' treatment for the first cycle, escalating to 10 then 14 days only if no grade 3 or 4 toxicity was encountered. Of 34 patients treated on this escalating schedule, 17 (50%) reached the full 14 day regimen. The others continued on a 7 day (six patients) or 10 day (five patients) schedule, or had been withdrawn before full escalation could occur. Inability to tolerate the full 14 day schedule correlated with age, performance status and number of previous treatments, but not with mild renal or hepatic dysfunction.

After this protocol modification, only two of 34 patients, one with severe renal impairment, developed grade 4 myelotoxicity, and treatment was generally well tolerated. The patient with renal impairment (glomerular filtration rate 14 ml $min^{-1}$) was also hypoalbuminaemic (29 g $l^{-1}$). She was given a test dose on day 1, following which total plasma etoposide pharmacokinetics was measured using a limited sampling strategy described elsewhere (Joel *et al.*, 1991*b*). A dose reduction was calculated and a further 6 days' treatment was given at 50 mg o.d./b.d. on alternate days. Further sampling on day 7 confirmed that drug accumulation had not occurred. However, despite this, grade 4 myelotoxicity developed from day 11, complicated by *Staphylococcus aureus* septicaemia, and the patient subsequently died.

### *Response in relation to toxicity and dose intensity*

Taking all 41 assessable patients (original + modified protocols), there is no correlation between the highest grade of myelotoxicity and the response to treatment (overall $\chi^2$ test, $P = 0.9$). Thus, responses and disease stabilisation were seen as commonly in patients who had only grade 0–1 myelotoxicity as in those showing significant myelotoxicity during treatment.

For patients on the modified protocol, there was a relationship between the ability to escalate to the full 14 day schedule and the subsequent response. One CR and two SD were seen among the 11 patients who could not tolerate the full 14 day regimen (response + SD = 21%), compared with one CR, seven PR and four SD among the 17 who could (response + SD = 70%). However, this difference might relate to the poorer performance status of the former group rather than to the lower dose intensity received.

### Discussion

This study demonstrates that oral etoposide has activity in relapsed ovarian cancer. In this population of patients with bulky and multiple sites of disease the objective response rate of 24% (95% confidence interval 12–41%) is encouraging. This experience is superior to that of Marzola *et al.* (1993), who recently reported only one PR among 17 patients using oral etoposide 50 mg o.d. for 21 days every 4 weeks. Their patients were more heavily pretreated (all ≥2 previous treatments), but of better performance status (all 0–1) and with a longer treatment-free interval (>6 months in 55%). It is possible that the lower dose intensity of the 50 mg o.d. schedule was a factor.

**Table IV** Characteristics of patients with severe first-cycle toxicity

| *Age* | *Performance status* | *Renal impairment* | *Hepatic impairment* | *Cycle 1 duration* | *Haemotological toxicity* | *Mucosal toxicity* | *Outcome* |
|---|---|---|---|---|---|---|---|
| 56 | 3 | – | + | 14 days | 4 | 4 | Toxic death |
| 67 | 3 | + + | – | 7 days | 4 | 0 | Toxic death |
| 47 | 3 | – | – | 14 days | 4 | 0 | Withdrawn (PD) |
| 56 | 2 | – | + | 14 days | 4 | 3 | Withdrawn (toxicity) |
| 76 | 1 | – | – | 14 days | 4 | 3 | Withdrawn (toxicity) |

The result of this study should be interpreted in the light of the published phase II studies of taxol in relapsed ovarian cancer (Einzig *et al.*,1992; McGuire *et al.*, 1989), in which a total of 70 patients have been treated with two complete and 16 partial responses (CR + PR 26%; 95% confidence intervals 16–38%). One of these studies excluded patients with more than one previous chemotherapy treatment. Data recently reported from the use of taxol in very heavily pretreated patients ($\geqslant 3$ prior regimens) throughout the USA suggested a response rate of 21%, with 19% SD (Trimble *et al.*, 1993).

Whether etoposide's activity is clinically useful depends on the balance of treatment-induced toxicity against anticancer activity, a judgement that must be made for individual patients. The consistent side effect of etoposide is alopecia which, for some but not all patients, is an important price to pay for a modest chance of benefit. For many patients this was the only significant side effect, but more worrying were the episodes of severe myelotoxicity, with two treatment-related deaths. All the affected patients had one or more risk factors: performance status $\geqslant 3$; moderate/severe hepatic or renal impairment, age over 75. The incidence of severe toxicity is minimised by avoiding patients with these risk factors and using the escalating schedule.

Haematopoietic growth factors were not used during this study, and three observations suggest that the routine use of granulocyte or granulocyte–macrophage colony-stimulating factor for 'poor-risk' patients would not have been helpful:

(1) The risk factors for toxicity appeared also to predict for failure to respond.
(2) Severe neutropenia, when it occurred, was accompanied by thrombocytopenia.
(3) Response did not correlate positively with myelotoxicity overall.

A policy of patient selection to avoid severe toxicity would therefore seem more appropriate than one of 'treat and rescue'. However, growth factors may, of course, be appropriate in cases of unexpected severe neutropenia.

Etoposide's oral bioavailability shows marked inter- and intra-patient variability (Harvey *et al.*, 1985). Once absorbed, it is largely bound to plasma proteins, with free drug being cleared by both renal and hepatic routes. There is therefore much potential net variability in the pharmacokinetics of the drug in cancer patients, who may have altered gastrointestinal function, hypoproteinaemia and hepatic or renal dysfunction. Pharmacokinetic monitoring and dose adjustment in our patient with renal impairment failed to prevent excessive toxicity, however hypoalbuminaemia with consequently reduced protein binding may have confounded the pharmacokinetic monitoring in this case.

The stimulus to the development of protracted oral etoposide schedules is the hypothesis that, in small cell lung cancer, a pharmacodynamic relationship exists between etoposide's anti-tumour activity and the duration for which plasma levels are maintained above a low threshold value in the region of $1\ \mu g\ ml^{-1}$ (Slevin, 1990). The schedule of 50 mg b.d. used in this study produces plasma etoposide $> 1\ \mu g\ ml^{-1}$ for a median of 14 h out of every 24 (Joel *et al.*, 1991*b*). However, the prolonged drug exposure provided in this study has not resulted in improved activity compared with previous reports of 3 or 4 day intravenous or oral schedules (Kuhnle *et al.*, 1988; Kavanagh *et al.*, 1989; Eckhardt *et al.*, 1990; Hillcoat *et al.*, 1985; Hansen *et al.*, 1990). There are several possible explanations for this:

(1) A 4 day schedule may be long enough to exploit fully any schedule dependency of etoposide in ovarian cancer, with no additional benefit from more prolonged scheduling.
(2) The patient population may be different: the largest and most optimistic study of intravenous etoposide was in patients with only one previous chemotherapy exposure (Kuhnle *et al.*, 1989).
(3) The variable oral bioavailability of etoposide may result in failure to reach the threshold concentration for activity in a proportion of the patients. Continuous ambulatory intravenous infusion would provide a superior, if less convenient, means of maintaining consistent prolonged low plasma levels (Greco *et al.*, 1992). Alternatively, etoposide phosphate, a water-soluble prodrug with improved oral bioavailability in animal studies, may provide a future solution to the problem of oral etoposide dosing.

In conclusion, oral etoposide has significant activity against relapsed and platinum-resistant ovarian cancer and bears comparison with the best alternative intravenous therapies. It should be employed with caution: patients who are elderly, of poor performance status or with moderate to severe hepatic or renal impairment may suffer severe myelotoxicity, not necessarily preventable by pharmacokinetic-guided dose adjustment, and are in any case unlikely to benefit from treatment. Conversely, relatively fit patients may benefit from this outpatient treatment, whose main toxicity when properly monitored is reversible alopecia. Continuous ambulatory intravenous infusion of etoposide is an alternative means of providing optimal prolonged-schedule therapy and is currently under investigation.

We are indebted to Rod Cummings, Catherine Oakley, Julie Clark, Uma Samasundrum, Rita Winright and Nancy Preston for the collection of data, and to the many doctors and nurses who have been involved in the care of the patients reported.

### References

BELINSON, J.L., PRETORIUS, R.G., MCCLURE, M. & ASHIKAGA, T. (1986). Hexamethylmelamine, methotrexate, 5-fluorouracil as second-line chemotherapy after platinum for epithelial ovarian malignancies. *Gynecol. Oncol.*, **23**, 304–309.

BENEDETI-PANICI, P., SCAMBIA, G., GREGGI, S., SALERNO, G., CENTO, R. & MANCUSO, S. (1990). Doxorubicin and cyclophosphamide alternated with bleomycin and mitomycin C as a second-line regimen in advanced ovarian cancer resistant to cisplatin-based chemotherapy. *Oncology*, **47**, 296–298.

CLARK, P.I., COTTIER, B., JOEL, S.P., THOMPSON, P.I. & SLEVIN, M.L. (1990). Prolonged administration of single-agent oral etoposide in patients with untreated small-cell lung cancer (meeting abstract). *Proc. Am. Soc. Clin. Oncol.*, **9**, 226 (abstract 874).

COLEMAN, R., CLARK, J., GORE, M., WILTSHAW, E., SLEVIN, M. & HARPER, P. (1989). A phase II study of mitozantrone in advanced carcinoma of the ovary. *Cancer Chemother. Pharmacol.*, **24**, 200–202.

COLEMAN, R., TOWLSON, K., WILTSHAW, E., SLEVIN, M., BLAKE, P., STEIN, R., COOMBES, R. & HARPER, P. (1990). Epirubicin for pretreated advanced ovarian cancer (letter). *Eur. J. Cancer*, **26**, 850–851.

ECKHARDT, S., HERNADI, Z., THURZO, L., TELEKES, A., SOPKOVA, B., MECHL, Z., PAWLICKI, M. & KERPEL-FRONIUS, S. (1990). Phase II clinical evaluation of etoposide as a second-line treatment in ovarian cancer. *Oncology*, **47**, 289–295.

EINZIG, A.I., WIERNIK, P.H., SASLOFF, J., RUNOWICZ, C.D. & GOLDBERG, G.L. (1992). Phase II study and long-term follow-up of patients treated with taxol for advanced ovarian adenocarcinoma. *J. Clin. Oncol.*, **10**, 1748–1753.

GRECO, F.A., JOHNSON, D.H. & HAINSWORTH, J.D. (1990). Chronic daily administration of oral etoposide. *Semin. Oncol.*, **17** (suppl. 2), 71–74.

GRECO, F.A., GARROW, G.C., JOHNSON, D.H., HANDE, K.R., STEIN, R.S. & HAINSWORTH, J.D. (1992). Prolonged continuous infusion of low dose etoposide in responsive neoplasms: preliminary phase I/II results (meeting abstract). *Proc. Am. Soc. Clin. Oncol.*, **11**, 115 (abstract 282).

HANSEN, F., MALTHE, I. & KROG, H. (1990). Phase-II clinical trial of etoposide administered orally in advanced ovarian cancer. *Gynecol. Oncol.*, **36**, 369–370.

HARVEY, V.J., SLEVIN, M.L., JOEL, S.P., SMYTHE, M.M., JOHNSTON, A. & WRIGLEY, P.F.M. (1985). Variable bioavailability following repeated oral doses of etoposide. *Eur. J. Cancer Clin. Oncol.*, **21**, 1315–1319.

HILL, B.T., WHELAN, R.D.H., RUPNIAK, H.T., DENNIS, L.Y. & ROSHOLT, M.A. (1981). A comparative assessment of the in vitro effects of drugs on cells by means of colony assays or flow microfluorimetry. *Cancer Chemother. Pharmacol.*, **7**, 21–26.

HILLCOAT, B.L., CAMPBELL, J.J., PEPPERELL, R., QUINN, M.A., BISHOP, J.F. & DAY, A. (1985). Phase-II trial of VP-16-213 in advanced ovarian carcinoma. *Gynecol. Oncol.*, **22**, 162–166.

JOEL, S., CLARK, P. & SLEVIN, M. (1991*a*). Renal function and etoposide pharmacokinetics: is dose modification necessary? (meeting abstract). *Proc. Am. Soc. Clin. Oncol.*, **10**, 103 (abstract 281).

JOEL, S.P., DOLEGA-OSSOWSKI, E., JONES, K., CLARK, P.I., JOHNSON, P. & SLEVIN, M.L. (1991*b*). The bioavailability of oral etoposide during prolonged oral administration and development of a limited sampling strategy for the estimation of AUC after an oral dose (meeting abstract). *Proc. Am. Assoc. Cancer Res.*, **32**, 178 (abstract 1061).

KAVANAGH, J.J., MORRIS, M., SMALLDONE, L., KWAN, J. & AMMERMAN, M.B. (1989). A randomized trial of carboplatin versus variably timed continuous infusion etoposide in refractory epithelial ovarian cancer (meeting abstract). *Proc. Am. Soc. Clin. Oncol.*, **8**, 163 (abstract 637).

KUHNLE, H., MEERPOHL, H.G., LENAZ, L., ACHTERRATH, W., POHL, J., ROBEN, S., ALFUB, S., PFLEIDERER, A. & KUHN, W. (1988). Etoposide in cisplatin-refractory ovarian cancer (meeting abstract). *Proc. Am. Soc. Clin. Oncol.*, **7**, 137 (abstract 527).

LEE, C.R., NICHOLSON, P.W., SOUHAMI, R.L., SLEVIN, M.L., HALL, M.R. & DESHMUKH, A.A. (1993). Patient compliance with prolonged low-dose oral etoposide for small cell lung cancer. *Br. J. Cancer*, **67**, 630–634.

MCGUIRE, W.P., ROWINSKY, E.K., ROSENSHEIN, N.B., GRUMBINE, F.C., ETTINGER, D.S., ARMSTRONG, D.K. & DONEHOWER, R.C. (1989). Taxol: a unique antineoplastic agent with significant activity in advanced ovarian epithelial neoplasms. *Ann. Intern. Med.*, **111**, 273–279.

MANETTA, A., MACNEILL, C., LYTER, J.A., SCHEFFLER, B., PODCZASKI, E.S., LARSON, J.E. & SCHEIN, P. (1990). Hexamethylmelamine as a single second-line agent in ovarian cancer. *Gynecol. Oncol.*, **36**, 93–96.

MARZOLA, M., ZUCCHETTI, M., COLOMBO, N., SESSA, C., PAGANI, O., D'INCALCI, M., CAVALLI, F. & MANGIONI, C. (1993). Low-dose oral etoposide in epithelial cancer of the ovary. *Ann. Oncol.*, **4**, 517–519.

MILLER, J.C. & EINHORN, L.H. (1990). Phase II study of daily oral etoposide in refractory germ cell tumours. *Semin. Oncol.*, **17** (suppl. 2), 36–39.

PATER, J.L., CARMICHAEL, J.A., KREPART, G.V., FRASER, R.C., ROY, M., KIRK, M.E., LEVITT, M., BROWN, L.B., WILSON, K.S., SHELLEY, W.E. & WILLAN, A.R. (1987). Second-line chemotherapy of stage III–IV ovarian carcinoma: a randomized comparison of melphalan to melphalan and hexamethylmelamine in patients with persistent disease after doxorubicin and cisplatin. *Cancer Treat. Rep.*, **71**, 277–281.

SIMON, R.M. (1989). Design and conduct of clinical trials. In *Cancer: Principles and Practice of Oncology*, 3rd edn., DeVita, V.T., Hellman, S. & Rosenberg, S.A. (eds) pp. 369–420.

SLEVIN, M.L. (1990). Low dose oral etoposide: a new role for an old drug? (editorial). *J. Clin. Oncol.*, **8**, 1607–1609.

SLEVIN, M.L., CLARK, P.I., JOEL, S.P., MALIK, S., OSBORNE, R.J., GREGORY, W.M., LOWE, D.G., REZNEK, R.H. & WRIGLEY, P.F.M. (1989*a*). A randomised trial to evaluate the effect of schedule on the activity of etoposide in small-cell lung cancer. *J. Clin. Oncol.*, **7**, 1333–1340.

SLEVIN, M.L., CLARK, P.I., JOEL, S.P., THOMPSON, P., TALBOT, D., PRICE, C. & WRIGLEY, P.F.M. (1989*b*). A randomised trial to examine the effect of more extended scheduling of etoposide administration in small cell lung cancer (meeting abstract). *Proc. Am. Soc. Clin. Oncol.*, **8**, 236 (abstract 921).

SUTTON, G.P., BLESSING, J.A., HOMESLEY, H.D., BERMAN, M.L. & MALFETANO, J. (1989). Phase II trial of ifosfamide and mesna in advanced ovarian carcinoma: a Gynecologic Oncology Group study. *J. Clin. Oncol.*, **7**, 1672–1676.

TRIMBLE, E.L., ADAMS, J.D., VENA, D., HAWKINS, M.J., FRIEDMAN, M.A., CANETTA, R., ONETTO, N., HAYN, R. & ARBUCK, S.G. (1993). Taxol in patients with platinum-resistant ovarian cancer (meeting abstract). *Proc. Am. Soc. Clin. Oncol.*, **12**, 261 (abstract 829).

WHO (1979). *WHO Handbook for Reporting Results of Cancer Treatment*. World Health Organization Offset Publication No. 48: Geneva.